# Long-term clinical and experimental/surface analytical studies of carbon/carbon maxillofacial implants

**DOI:** 10.1186/s40902-015-0031-3

**Published:** 2015-10-01

**Authors:** György Szabó, József Barabás, Sándor Bogdán, Zsolt Németh, Béla Sebők, Gábor Kiss

**Affiliations:** 1 Department of Oral and Maxillofacial Surgery and Dentistry, Semmelweis University, Mária utca 52, Budapest, 1085 Hungary; 2 Department of Atomic Physics, University of Technology and Economics, Budafoki út 8, Budapest, 1111 Hungary

**Keywords:** Carbon implant, Complication, Reconstruction, Mandible

## Abstract

**Background:**

Over the past 30–40 years, various carbon implant materials have become more interesting, because they are well accepted by the biological environment. The traditional carbon-based polymers give rise to many complications. The polymer complication may be eliminated through carbon fibres bound by pyrocarbon (carbon/carbon). The aim of this study is to present the long-term clinical results of carbon/carbon implants, and the results of the scanning electron microscope and energy dispersive spectrometer investigation of an implant retrieved from the human body after 8 years.

**Methods:**

Mandibular reconstruction (8–10 years ago) was performed with pure (99.99 %) carbon implants in 16 patients (10 malignant tumours, 4 large cystic lesions and 2 augmentative processes). The long-term effect of the human body on the carbon/carbon implant was investigated by comparing the structure, the surface morphology and the composition of an implant retrieved after 8 years to a sterilized, but not implanted one.

**Results:**

Of the 16 patients, the implants had to be removed earlier in 5 patients because of the defect that arose on the oral mucosa above the carbon plates. During the long-term follow-up, plate fracture, loosening of the screws, infection or inflammations around the carbon/carbon implants were not observed. The thickness of the carbon fibres constituting the implants did not change during the 8-year period, the surface of the implant retrieved was covered with a thin surface layer not present on the unimplanted implant. The composition of this layer is identical to the composition of the underlying carbon fibres. Residual soft tissue penetrating the bulk material between the carbon fibre bunches was found on the retrieved implant indicating the importance of the surface morphology in tissue growth and adhering implants.

**Conclusions:**

The surface morphology and the structure were not changed after 8 years. The two main components of the implant retrieved from the human body are still carbon and oxygen, but the amount of oxygen is 3–4 times higher than on the surface of the reference implant, which can be attributed to the oxidative effect of the human body, consequently in the integration and biocompatibility of the implant. The clinical conclusion is that if the soft part cover is appropriate, the carbon implants are cosmetically and functionally more suitable than titanium plates.

## Background

During recent years, various carbon implant materials have become of considerable interest in view of the fact that they are well tolerated by the biological environment [1]. The traditional carbon-based polymers (hip and knee prostheses and heart valves) give rise to certain complications because of the polymer matrix [2], such as material fatigue and fractures. These complications may be eliminated through the use of carbon fibres bound by pyrocarbon (carbon/carbon, C/C) [3].

The mechanical properties of C/C composites are very close to those of human bones [4]. This is a major advantage as compared with different metals, and especially titanium implant materials. The screws employed to fix the metal to the bone impose a much greater load on the metal-bone connection than in the case of a C/C and bone [5, 6].

During the manufacturing of C/C composites, layers of carbon fibre fabric are combined together with a carbonaceous material to give the composite the desired shape. After multiple heat treatments and densification processes, a lightweight, yet mechanically stable composite is produced, which is almost entirely composed of carbon. The bonding and densification can be achieved with the aid of different processes and precursor materials [7]. Such processes include chemical vapour deposition or chemical vapour infiltration. The composite produced is covered by pyrolytic carbon resulting from the thermo cracking of different carbonaceous materials used during the manufacturing. The biocompatibility of carbon materials, and especially carbon fibres and pyrolytic carbon-covered C/C composites, has been studied extensively in recent decades, and they are now widely accepted as biocompatible materials [8–12].

From a maxillofacial surgical aspect, relatively few C/C modules are available [13–15]. Russian and Polish authors have utilized such material in the form of powder and solid bodies for the filling of jawbone cysts and for the replacement of the alveolus, the facial bone or the jawbone. The failure that occurred in approximately one sixth of the cases was not a result of spontaneous rejection of the C/C prostheses, but developed in consequence of various technical problems that arose in the course of the implantation. The main problems involved the insufficient covering of the soft parts or opening of the wound because of the strain in it. By contrast, cranial replacement proved 100 % successful [16]. It should be mentioned, of course, that cranial replacement is a much simpler operation than the functional and aesthetic restoration of the jawbones.

During the period between 2002 and 2005, we had the possibility to make use of C/C material for purposes of jawbone replacement, and the more than 10 years that has subsequently elapsed is sufficient for us to draw long-term consequences.

A number of questions arise when the effectiveness of medical implants is evaluated. Perhaps the most important of these are as follows:

- What is the functional and aesthetic result? Did the implantation achieve the aim?
- What are the effects of the implant on its immediate and more distant environments? (Is there any systemic or local toxic effect?)
- What is the effect of the organism on the implant? How and to what extent can the implant be damaged by the loading imposed on it because of its function and by the aggressive action of the organism?

The aim of the present paper is to provide answers to these questions and to present the possibilities of the long-term application of certain carbon fibre-reinforced carbon (C/C) composites in maxillofacial surgery.

## Methods

### Patients and implantation

Between 2002 and 2005, implants made from Carbulat™ were utilized for purposes of jawbone replacement in 16 patients (6 women and 10 men; average age 41 years) (Table 1). In 2002, Carbulat™, which features on the lists of the U.S. Food and Drug Administration and of the WHO of the substances that may be implanted into the human organism, was licensed for use in oral surgical investigations at the Department of Maxillofacial and Oral Surgery and Dentistry at Semmelweis Uinversity; in consequence of the positive results, this licence was extended in 2003.The experimental research reported in the manuscript was performed with the approval of the Ethical Committe of the Semmelweis University.

**Table 1:** Clinical results

Diagnosis	No. of patients	Successful implantations
Mandibular cyst	4	2
Augmentation	2	2
Replacement after tumour surgery	10	7
Total	16	11

In the majority of the cases (10 patients), an extensive mandibular tumour had been removed, but for various reasons ‘living bone’ could not be used for replacement primarily. The excluding factors included the previous intensive cytostatic treatment (Ewing’s sarcoma in the mandible), the rejection of the previous replacement with fibula or the fact that the tumour had been so extensive that it was removed purely for palliative reasons (e.g. a very large osteosarcoma). In 4 cases, C/C granulate was utilized for filling after the removal of a mandibular cyst, and in 2 cases, a Carbulat™ plate was used for mandibular augmentation.

**Fig. 1:**
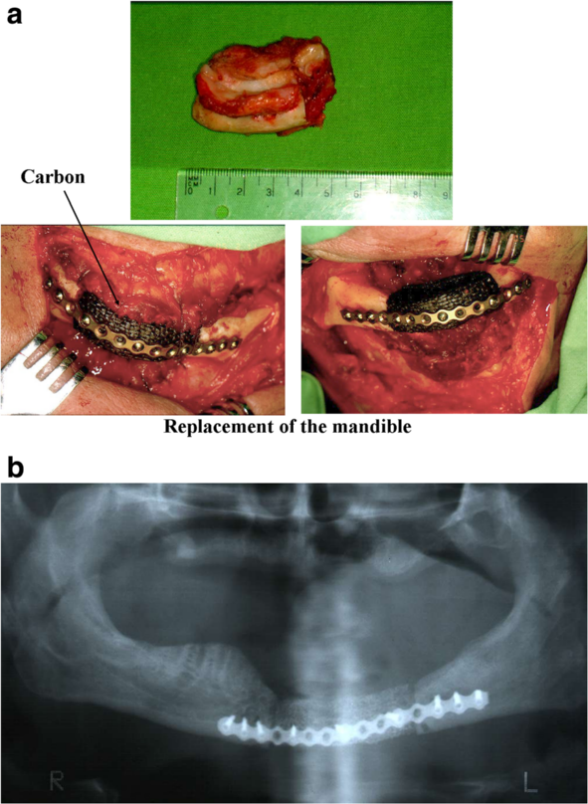
Compact implants with volumes corresponding to the body of the mandible. **a** Compact Carbulat™ implant, after resection of a gingival carcinoma (post. irrad.). **b** Panoramic X-ray after the surgery. The density of the Carbulat™ implant is very close to those of human bone
- A 2-mm-thick net that can be fitted (screwed) to the external surface of the mandible and which follows the curvature of the mandible (Fig. 2).

The Carbulat™ implants, containing 99 % carbon, were produced by Ametist Goldy Management Group L.L.C. We applied them in basically three forms:

- A granulate measuring 500–1000 μm (for the filling of cystic cavities)
- Compact implants with volumes corresponding to the body of the mandible (approximately 15 mm thick) (Fig. 1)

**Fig. 2:**
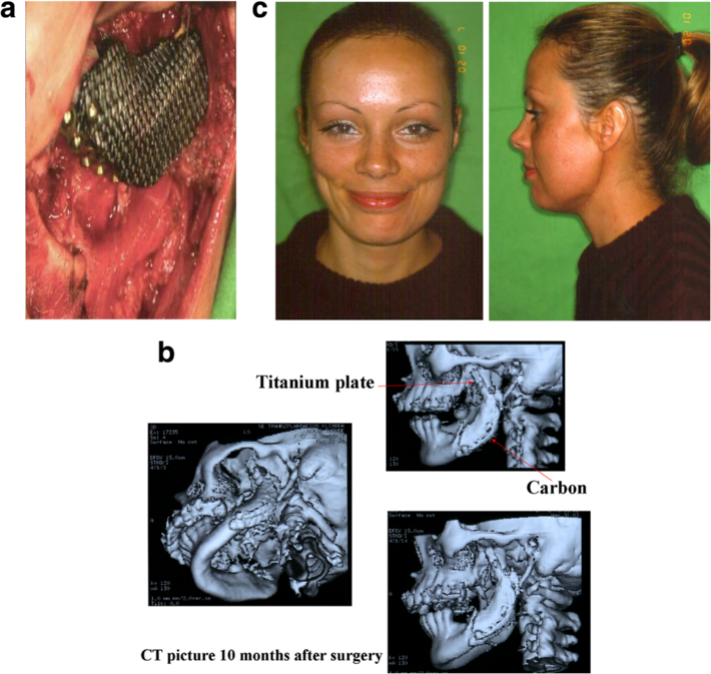
A 2-mm-thick net. **a** Resection of the mandible (synovial sarcoma) 2-mm-thick carbon/carbon net is screwed to the external surface of the mandible, follows the curvature of the bone. **b** CT picture after surgery. **c** 3 years after surgery

The mandibular replacement was carried out by the customary method: before resection of the bone (if that was not made impossible by the tumourous lesion), a 1.8-mm-thick titanium plate suitable for osteosynthesis was screwed to the external side of the mandible. This plate ensured that the mandible remained in its original position after the resection. This was followed (with the plate left in place) by removal of the diseased bone part. The pre-prepared Carbulat™ piece was next fitted closely at the site of the resected bone and screwed to the titanium plate. The correction was performed with a diamond cutter under physiological saline solution in a vessel suitable for this purpose, so that the black powder produced during the cutting should not contaminate the environment. In the case of the Carbulat™ net, the titanium plate could be removed in most cases if the implant could be fitted securely with screws to the distal and proximal bone parts. The most important task after the insertion of the Carbulat™ was to enclose the implant from the directions of both the face and the oral cavity with the thickest possible layer of soft parts. In the cases of patients who had undergone surgery and irradiation on a number of occasions, strain-free closure generally involved difficulty, and therefore demanded particular care.

**Fig. 3:**
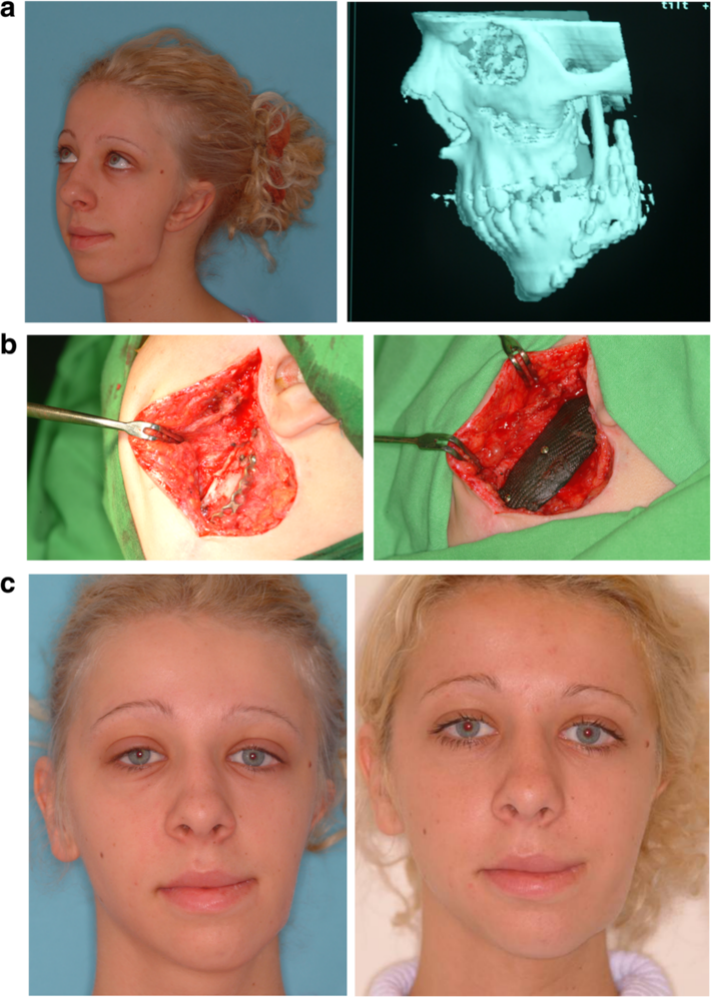
The bone and soft part deficiency causing the persisting facial asymmetry was corrected with a Carbulat™ net. **a** Hemifacial microsomia, 12 years after double rib-transplantation. **b** The Carbulat™ prefabricated plate screwed on the bone. **c** The patient before and 1 week after the surgery

Compact Carbulat™ was applied in 2 patients, and mandibular bone was supplemented with a Carbulat™ net in 8 cases. The Carbulat™ net was used for augmentation purposes in 2 cases. In 1 patient, the facial asymmetry was corrected with a 5 × 2.5 cm net 5 years after the exstirpation of a tumour affecting the mandible. In the other case (hemifacial microsomia), rib transplantation had already been performed on two occasions in order to supplement the ascending branch of the mandible. Then, 10 years later, when the patient was 18 years old, the bone and soft part deficiency causing the persisting facial asymmetry was corrected with a Carbulat™ net prepared in appropriate size. This was necessary as the patient did not agree to undergo further autologous bone transplantation (Fig. 3).

### Long-term follow-up studies

For study of the long-term effects of the interaction between an implant and the human body, the surface morphology and the average surface composition of an implanted Carbulat™ implant (retrieved after 8 years) and a sterilized but not implanted Carbulat™ reference implant were compared. After 8 years, the alloplastic material was removed from the above 18-year-old asymptomatic patient, and the composite implant was replaced with autologous bone tissue from the iliac crest.

### Sample preparation

Before the measurements, the implants were cut into smaller pieces with a saw that had been cleaned with ethanol, and the pieces were dried in the air. All the tools used to mount the samples on sample holders were cleaned with an ethanol ultrasonic bath.

Three types of samples were investigated:

- Unimplanted (reference) implants which were examined immediately after being unpacked from their sterile packing.
- Pieces of the implant retrieved from the human body after 8 years. Some of these were cleaned to remove the residual soft tissue before investigations of the surface morphology and composition. This cleaning process was carried out with Enzyrim-Oss (Arte-Copia, Switzerland) dissolved in tap-water at a concentration of 20 ml/l, this material being used to remove soft tissues from hard tissues in forensic science [17].
- Other pieces of the implant retrieved from human body after 8 years were investigated without the enzymatic treatment in order to verify that the enzyme solution only removes the residual soft tissues from the surface and does not alter the structure or the surface composition of the C/C composite.

### Experimental methods applied

The surface morphology of the implants was investigated with a Philips XL30 scanning electron microscope (SEM), and a Bruker QUANTAX 200 energy dispersive spectrometer (EDS measurements) with an XFlash 5010 detector attached to an FEI Inspect S50 SEM was used for the measurement of the average surface composition.

#### Surface morphology investigations

The surface morphology of the samples was observed with the aid of secondary electron images of the surface. In the secondary electron mode, the surface is scanned with a high-energy electron beam (in our case 20 keV) and the intensity of the low-energy (<50 eV) electrons leaving the surface is measured. The resulting images have topographical contrast, and the morphology of the surface can therefore be studied.

#### Surface composition investigations

EDS measurements were carried out to obtain quantitative information on the surface composition. During these measurements, the spectrum of the characteristic X-rays induced by the high-energy electron beam is detected. The acceleration voltage applied in our EDS measurements (20 kV) gives an informal depth of about 4–5 μm in pure carbon [18]. The spectra were evaluated with Bruker ESPRIT 1.9 software, which uses the standardless P/B-ZAF (peak to background ZAF) method.

Besides the EDS measurements, back-scattered electron (BSE) images were also taken of the surface of the samples. In these images, areas with higher average atomic numbers appear brighter than those with lower ones.

## Results

### Fate of surgical implantations

Of the 4 mandibular cyst fillings, 2 were successful. In the 2 successful cases, healing was ensured by the good closure of the mucosa. In both of the 2 cases that failed (in each of which the upper and lateral bony wall of the cyst was missing), the wound opened after a few days and it was therefore necessary to remove the implanted material.

Both augmentation cases proved successful. It was possible to cover the implants with a thick layer of soft parts (skin and muscle), and there was therefore no obstacle to healing.

It was necessary to remove the implant from 3 of the 10 tumourous patients, in each of the 3 cases because of intraoral wound opening. The patients had previously undergone surgery and radiation treatment on a number of occasions. The intraoral suture was strained, and as a consequence of the previous events, the mucosal blood supply had deteriorated.

**Fig. 4:**
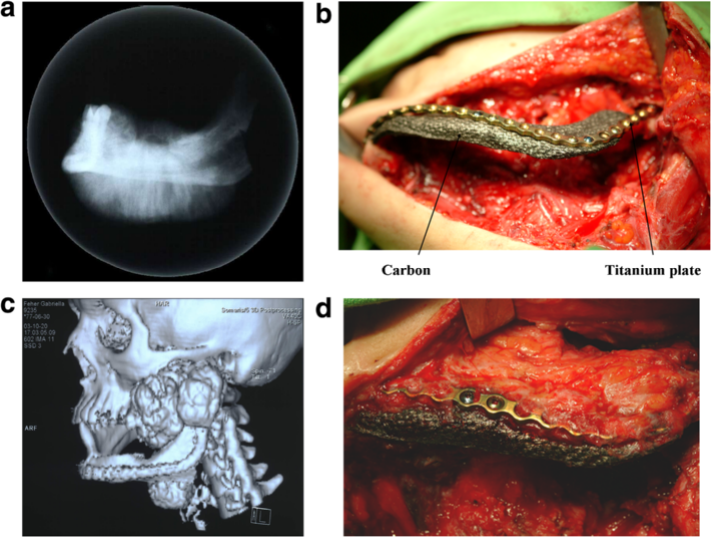
The Carbulat™ plate had to be removed because of tumour recurrence. **a** X-ray picture of a huge osteosarcoma of the mandible. Very characteristic sclerotizing form. **b** Carbulat™ and titanium plate reconstruction. **c** 8 months after the first surgery the relapsed tumour (CT picture). **d** After removal the relapsed tumour the Carbulat plate. It is visible that the Carbulat™ plate integrated extremely well into the tissues

In those cases when the Carbulat™ plate had to be removed because of tumour recurrence or in a planned manner because of the later autologous bone transplantation, it was seen that the Carbulat™ had integrated extremely well into the tissues (Fig. 4). It was also visible that black carbon particles had migrated into the soft parts surrounding the plate. Inflammatory and histological changes of any other type could not be discerned around the fine particles either clinically or histologically. In one case, autologous bone transplantation was performed after more than 8 years. However, in that case too there were no visible changes around the implant apart from the black discolouration mentioned above. Histological examinations were performed on the material containing the carbon particles. The histologist’s report contained the following finding: “..... In the deeper regions, scar connective tissue can be seen, in which a foreign body is to be found in places, in the form of small black particles. Individual lymphocytes occur in the vicinity of these particles, but no appreciable inflammatory changes are visible, and no giant cell or granuloma formation can be observed”.

Of the 16 implants, therefore, 5 had to be removed before time because of the intraoral wound opening. Two implants were removed in order to carry out a planned bone implantation or in consequence of tumour recurrence. Three patients died between 3 and 5 years post-implantation with the implant still in place. Six implants currently remain in place and functioning: 2 cysts, 2 augmentations and 2 reconstructions following tumour surgery. The patients did not agree to the recommended bone implantation as they were satisfied with the quality of life ensured by the implant.

### Follow-up investigations of implants

**Fig. 5:**
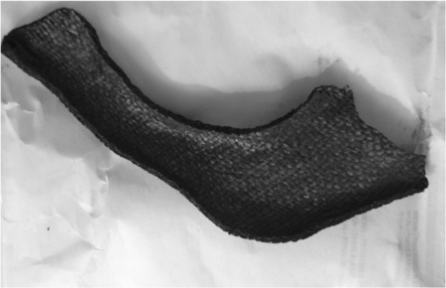
Reference implant inner side

Figure 5 shows the inner side of the reference implant that is closer to the oral cavity, immediately after the implant had been unpacked from the sterile packaging. During the implantation, this side is placed on the ends of the mandible and fixed with screws, substituting the missing part of the mandibular bone. The other side of the implant is referred to below as the outer side.

#### Morphology and structure of the implants

**Fig. 6:**
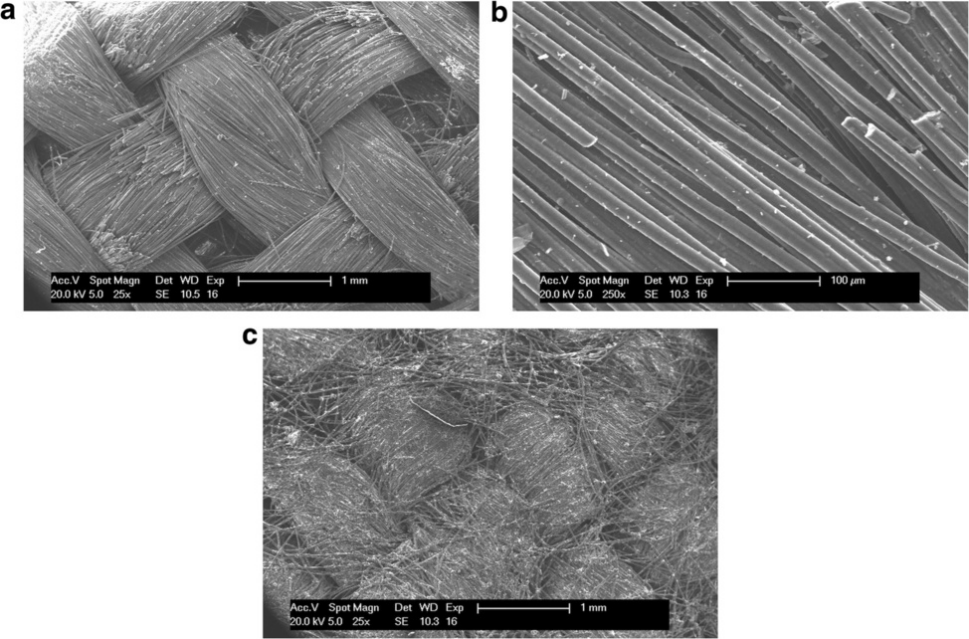
Scanning electron microscope investigation of the reference implant. **a** The outer side, magnification ratio ×25. **b** Outer side, magnification ratio ×250. **c** Inner side, magnification ratio ×25

On the outer surface of the reference implant (Fig. 6a), the fibres are arranged in bunches approximately 1 mm wide and perpendicular to each other. The manufacturer of the carbon fabric states that each bunch consists of around 6000 fibres aligned in parallel (Fig. 6b). Figure 6c shows the structure typical of the inner surface of the reference implant. The perpendicular bunches can be recognized, but the fibres present on the surface are less ordered than on the other side of the implant.

**Fig. 7:**
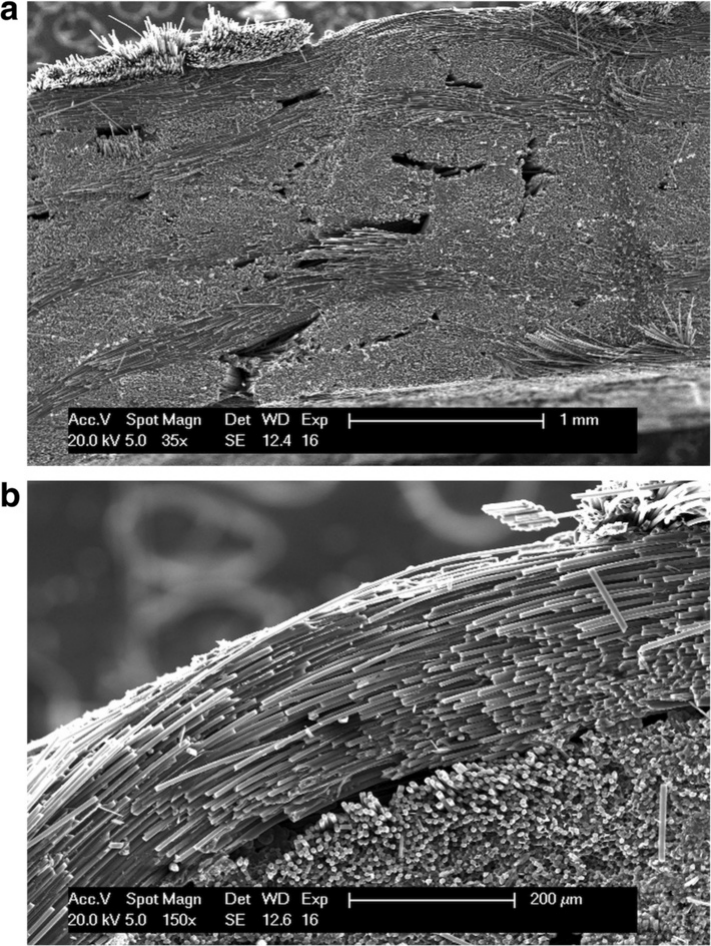
Cross section of the reference implant. **a** The whole cross section, magnification ratio ×25. **b** Near-surface region, magnification ratio ×150

Figure 7a illustrates the cross section of the reference implant, revealing the carbon fibre bunches perpendicular to each other with relatively small cavities between them. In the high-magnification image of the near-surface region of the implant (Fig. 7b), bunches headed to the direction of the cut and bunches perpendicular to it are also visible. A layer different from the fibre structure can not be observed on the surface of the implant or between the bunches.

**Fig. 8:**
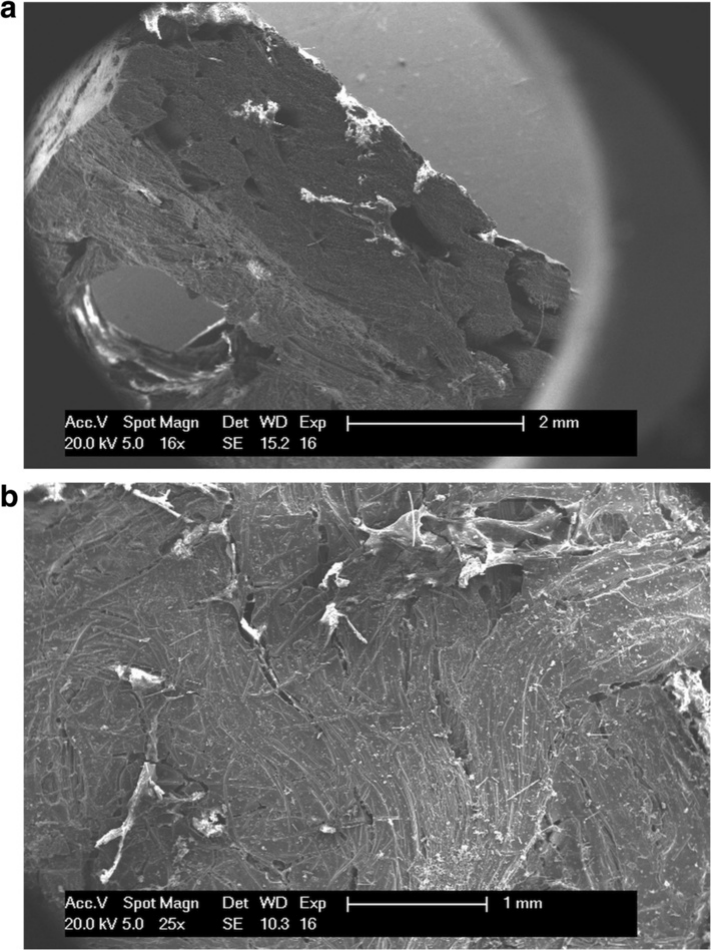
Scanning electron microscope images of the implant retrieved from the human body, before the enzyme treatment. **a** Cross section, inner side, magnification ratio ×16. **b** Surface of the outer side, magnification ratio ×25

Figure 8 shows the cross section: inner (a) and outer (b) side of the implant retrieved from the human body after 8 years, but before the enzyme treatment. During the implantation period, the implant was fixed to the mandible with screws. One of these screws passed through the hole seen in the Fig. 8a. The image of the cross section shows that the cavities between the carbon fibre bunches are larger than those in the reference implant. The white areas are non-conducting regions, which are charged with the scanning electron beam during the measurement. These regions probably consist of residual human soft tissues, which partially cover the surface of the implant and penetrate into the bulk material between the bunches and the implant surface. The residual tissues conceal the morphology of the surface on both sides of the implant.

**Fig. 9:**
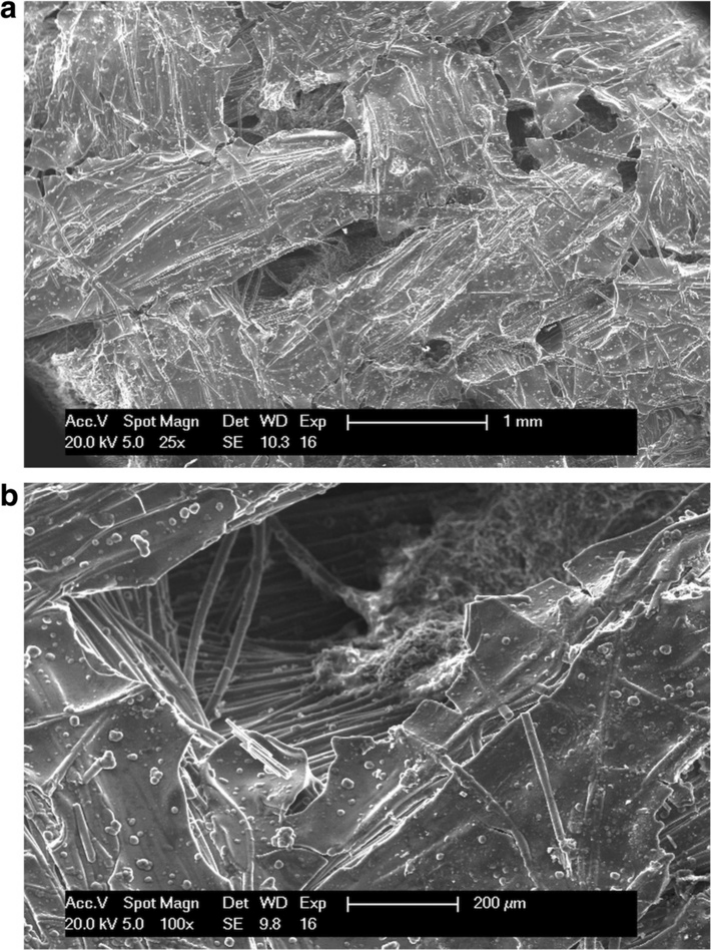
Scanning electron microscope images of the implant retrieved from the human body after the enzyme treatment (outer side). **a** Magnification ratio ×25. **b** Magnification ratio ×100

The implant retrieved from the human body was treated with an enzyme solution at 37 °C for 5 days in order to remove the remaining human soft tissues from the surface and thereby permit the study of the surface morphology. The inner and outer surfaces of the enzyme-treated implant proved to be very similar (Fig. 9).

**Fig. 10:**
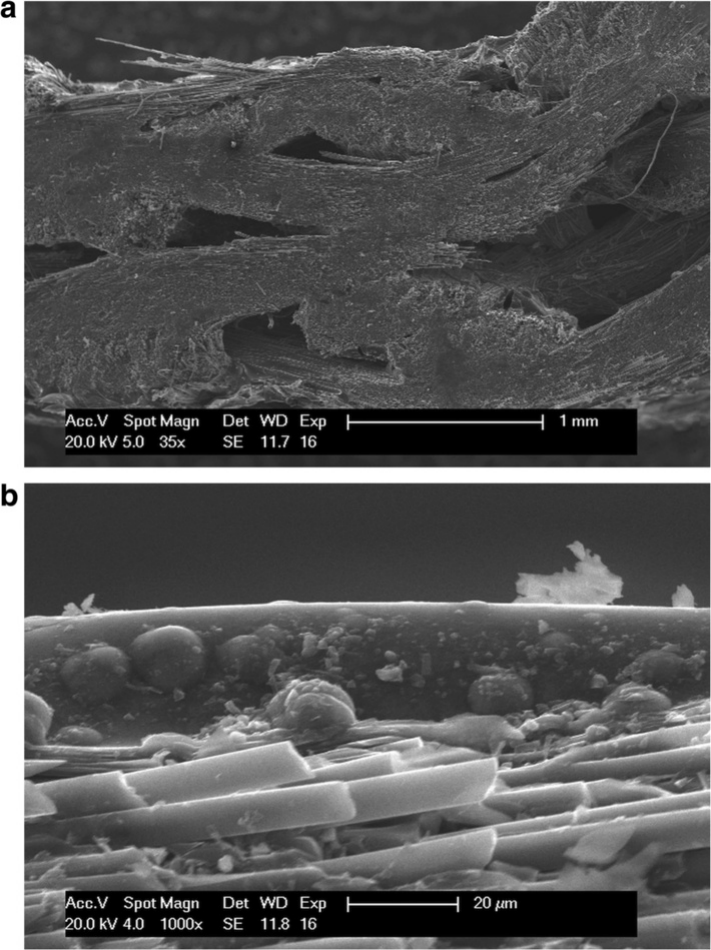
Scanning electron microscope images after enzyme treatment. **a** Cross section, magnification ratio ×35. **b** Near-surface region, magnification ratio ×1000

SEM images of the cross section and the outer surface of the implant can be seen in Fig. 10. In contrast with the images made before the enzymatic treatment, white spots can no longer be detected either on the surface or in the cross section. This tends to confirm the assumption that the white regions were images of the residual soft tissues, and that these were removed from the implant by the enzymatic treatment.

In the low-magnification image of the cross section (Fig. 10a), the cavities between the bunches are visible and are observed to be considerably larger than in the case of the reference implant (Fig. 7a) or before the enzyme treatment (Fig. 8a). Figure 10b illustrates the cross section of the near-surface region at high magnification. The surface is covered by a 15–17-μm-thick layer. This surface layer is also observed in the high-magnification images of the cross section of the implant retrieved from the human body, but prior to enzyme treatment. Just as in the case of the reference implant, a layer different from the fibre structure can not be observed between the carbon fibre bunches.

#### Thickness of the carbon fibres in the implants

In both the reference implant and the implant retrieved from the human body after 8 years, the measured thickness of the carbon fibres in the bunches was between 5 and 7 μm, in good agreement with the specification of the manufacturer of the carbon fabric. This suggests that the thickness of the carbon fibres did not change during the 8 years that the implant spent in the human body.

#### Surface composition of the implants

**Fig. 11:**
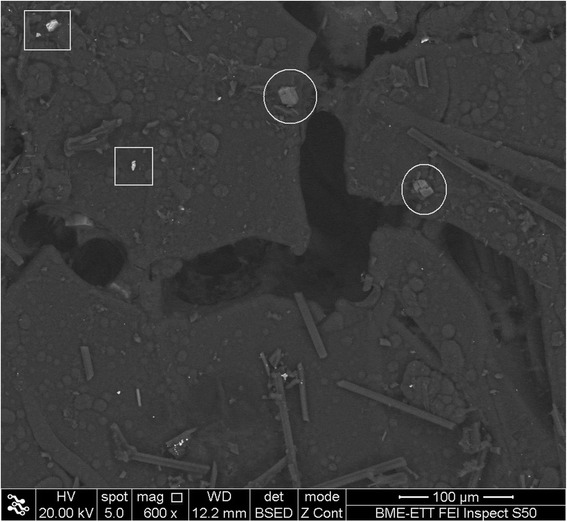
Back-scattered electron image of the surface, retrieved from the human body after enzyme treatment. Different brightness means difference in the average atomic number. The formations marked with *white rectangles* are iron rich, and the formations marked with *circles* are calcium-rich particles

In order to investigate the potential interactions between the implant and the human body, the average surface compositions of the reference implant and the implant retrieved from the human body were determined by the EDS method. The first two rows in Table 2 list the average surface compositions of the two sides of the reference implant. Besides the two main components, carbon (~97 at.%) and oxygen, small amounts of sodium, chlorine and zirconium were also detected. Crystals of sodium chloride (not shown) were seen in higher abundance on the inner side. Zirconium is added to the material of the implant during the manufacturing process in order to make the implant visible in X-ray or CT images. The BSE images (Fig. 11) and EDS measurements revealed that the zirconium was located between the bunches throughout the whole volume of the implant.

**Table 2:** Average surface composition (at.%) on both sides of the reference and the retrieved implants treated with the enzyme solution according to the EDS measurements

Sample	C (at.%)	O (at.%)	Na (at.%)	P (at.%)	S (at.%)	Cl (at.%)	Ca (at.%)	Fe (at.%)	Zr (at.%)
Reference outer side	97.4	2.6	^a^	^b^	^b^	^a^	^b^	^b^	^a^
Reference inner side	97.0	2.3	0.4	^b^	^b^	0.3	^b^	^b^	^a^
Retrieved outer side	92.7	7.3	^b^	^a^	^a^	^b^	^a^	^a^	^b^
Retrieved inner side	89.5	10.5	^b^	^a^	^a^	^b^	^a^	^a^	^b^

^a^The element is detectable as trace element, but the quantification is not possible (< 0.1 at.%)

^b^Element cannot be detected

Since the residual human soft tissues partially covered the surface of the implant retrieved from the human body, it was subjected to enzyme treatment before determination of the average surface compositions of the two sides. As the informal depth of EDS measurements on pure carbon with the parameters used is ~4.5 μm, only the average surface compositions of the 15–17-μm-thick surface layers on the two sides of the retrieved implant were determined. The measured compositions are to be found in rows 3 and 4 in Table 2.

Figure 11 presents an BSE image of the surface of the implant retrieved from the human body and then subjected to enzyme treatment. The brightness of a given area in an BSE image depends on the average atomic number and therefore on the composition. The recorded images revealed that the brightness of the image was homogeneous and identical to that of the fibres under this layer; the average atomic number of the layer was therefore laterally homogeneous and identical to the average atomic number of the carbon fibres.

## Discussion

A number of questions were raised in the Introduction. We are now in a position to give answers to these questions as concerns the C/C implants employed in the present investigation.

### Clinical experience

In connection with the first question, it may be stated that, in the cases (11 of the 16) when the Carbulat™ did not have to be removed before time, the result was very good from both a functional and an aesthetic aspect. When removal of the implant did become necessary before time, this was never due to any problem with the Carbulat™ as a material, but rather for various technical reasons: scar tissue with a poor blood supply, wound strain or wound opening. When the aesthetic results were compared with those achieved after the use of titanium reconstruction plates, the tissues proved to be better integrated around the Carbulat™ and the replacement with this material gave a better resemblance to the original shape and volume of the mandible.

As concerns, the question of systemic toxicity, it must be reiterated that the findings of the investigations performed to satisfy the demands of the U.S. Food and Drug Administration were satisfactory and carbon is not known to have a toxic effect [2]. In accordance with this, our regular laboratory examinations carried out over a period of several years following the implantation have never indicated the existence of even the slightest toxic effect in any of our cases.

From the aspect of the immediate environment of the implants, we conducted macroscopic and microscopic examinations on two implants, which had functioned in human organisms for 8 months and 8 years, respectively (Figs. 4 and 8). These had to be removed because of tumour recurrence or because implantation with autologous bone was planned. Macroscopically, it could be observed that a thin membrane similar to the periosteum had formed above the Carbulat™, and we consider this to be a sign of tissular integration. After removal of the implants, samples taken from this membrane and the adjacent tissues were examined histologically (data not shown). The results excluded the presence of any type of tissue damage or inflammation. It should be mentioned that we have observed a similar “periosteum” on the removal of alumina oxide or titanium implants, the difference being that the membrane adhered better to the Carbulat™ and was found clininally to be better integrated to it.

Our examinations to date have revealed black carbon particles only in the immediate vicinity of the plate; we have never observed them farther away (Fig. 4d). We consider that these black carbon particles may be formed for two reasons. The edges of the carbon are abraded in the course of the drilling of the plate, and further degradation results in the small particles. Alternatively, the organism itself breaks down the Carbulat™ to a slight extent, and these particles are the products of the breakdown.

To determine how the long-term exposure to the harsh environment of the human organism changes the structure and morphology of the implants, we set out to study this question with SEM and EDS.

### Surface morphology

The 5–7-μm-thick carbon fibres on the inner and outer surfaces of the reference implant (Fig. 6) were found to be ordered in bunches that were approximately 1 mm apart and perpendicular to each other, the fibres on the inner surface proving to be somewhat less ordered. This latter is probably due to damage inflicted during the retrieval of the implant, this process distorting the manufactured form.

Both the inner and the outer surface of the implant retrieved from the human body were covered by a 15–17-μm-thick cracked layer, below which the carbon fibres were still visible. As our measurements indicated that the thickness of the carbon fibres (5–7 μm) did not change during the 8-year period spent in the human body, the material constituting this surface layer presumably does not originate from the carbon fibres. A possible source is the pyrolytic carbon introduced to the implant during the manufacturing.

Cavities can be found between the perpendicularly oriented bunches in the implants. The cavities present in the implant retrieved from the human body were larger than those present in the reference implant. In the images of the retrieved implant (Fig. 10), human soft tissue can be seen to have penetrated through the space between the carbon fibre bunches. This supports the observation previously reported by other authors that the morphology of the surface of an implant plays an important role in the growth rate and sticking of the tissues, and consequently in the integration and biocompatibility of the implant [2, 9, 10, 19, 20].

### Surface composition of the C/C composite implant

Besides the two main components (carbon and oxygen), small quantities of sodium, chlorine and zirconium were detected on the surface of the reference implant (Table 2). The fact that the oxygen content on the surface of the implant retrieved from the human body was 3–4 times higher than that on the surface of the reference implant can be attributed to the oxidative effect of the human body. As a result of the interactions with the human organism, sodium, chlorine and zirconium were no longer detectable on the surface of the retrieved implant. This can be explained by the transport of these elements into the organism, or by the presence of a new surface layer that was thicker than the informal depth of the measurement method. Phosphorus, sulphur, calcium and iron were detected as trace elements on the surface of the implant retried from the human body (Table 2). Their presence can possibly be ascribed to their transport from the human organism: sulphur and phosphorus are present in proteins, calcium and phosphorus are present in bones, and the blood contains iron. Calcium and iron-rich particles were additionally detected on the surface of the retrieved implant (Fig. 11). The calcium-rich particles might be deposited on the surface during the removal of the screws fixing the implant to the mandible. The iron-rich particles could originate from the tools used during the removal surgery and the preparation of the sample for the measurements.

The effects of the human organism on the composition of the surface indicate that a slight extent of material transport occurs between the implant and the surrounding human tissues. It might prove beneficial to prevent this completely through the application of a stable and biocompatible surface thin layer.

## Conclusions

The clinical conclusion is that, if the soft part cover is appropriate, the carbon implants are cosmetically and functionally stable. The width of the carbon fibres (5–7 μm) building up the implants was not changed during the interaction with the human body. The structure and the morphology of the implants were not altered notably by the human body.

Further research is required to establish the effect of the findings presented here on the life quality of patients with pyrolytic carbon-covered C/C composite implants. A possible solution to enhance the long-term biocompatibility properties of the implants is the application of a thin surface layer of a biocompatible and inert material.

## Consent

Written informed consent was obtained from the patient for the publication of this report and any accompanying images.
